# Printable Single‐Ion Polymer Nanoparticle Electrolytes for Lithium Batteries

**DOI:** 10.1002/smsc.202300235

**Published:** 2024-01-14

**Authors:** Antonela Gallastegui, Rafael Del Olmo, Miryam Criado‐Gonzalez, Jose Ramon Leiza, Maria Forsyth, David Mecerreyes

**Affiliations:** ^1^ POLYMAT and Applied Chemistry Department University of the Basque Country UPV/EHU Avenida Tolosa 72 20018 Donostia‐San Sebastian Gipuzkoa Spain; ^2^ Institute for Frontier Materials and ARC Industry Training Transformation Centre for Future Energy Storage Technologies (StorEnergy) Deakin University Burwood Victoria 3125 Australia; ^3^ IKERBASQUE Basque Foundation for Science Bilbao 48009 Spain

**Keywords:** additive manufacturing, lithium batteries, polymer electrolytes, polymer nanoparticles, 3D printing

## Abstract

New material solutions are searched for the manufacturing and safety of current batteries. Herein, an extrusion printable polymer separator for lithium batteries based on single‐ion polymer electrolytes is presented. The polymer electrolytes are based on methacrylic polymeric nanoparticles (NPs) functionalized with a lithium sulfonamide group combined with different organic plasticizers such as sulfolane and carbonates. The synthesis of the polymer NPs is carried out by emulsion copolymerization of methyl methacrylate and lithium sulfonamide methacrylate in the presence of a crosslinker, resulting in particle sizes of less than 30 nm, as shown by electron microscopy. Then polymer electrolytes are prepared by mixing polymer NPs with varying lithium sulfonamide content and different plasticizers such as carbonates and sulfolane. The polymer electrolytes show ionic conductivities between 2.9 × 10^−4^ and 2.3 × 10^−5^ S cm^−1^ at 85 °C with the highest values for the small‐sized NPs with the highest lithium content. As a proof‐of‐concept application, layer‐by‐layer printing of a sulfolane‐based polymer electrolyte is evaluated via direct ink writing directly onto classic battery electrodes. The electrochemical characterization of the printed solid electrolyte indicates favorable properties, ionic conductivity, lithium transfer number, electrochemical stability window, and cyclability in lithium symmetrical cells, to be used in lithium batteries.

## Graphical abstract

This article shows an extrusion printable polymer separator for lithium batteries based on single‐ion polymer electrolyte. Methacrylic polymeric nanoparticles of 25 nm functionalized with a lithium sulfonamide group were combined with different organic plasticizers such as sulfolane and carbonates and tested in solid‐state batteries.

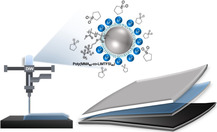

## 1 Introduction

The use of solid electrolytes in all solid‐state batteries (ASSBs) is increasingly being investigated due to the safety requirements and flammability issues of current liquid electrolytes.^[^ ^1^, ^2^ ^]^ Among the different types of solid electrolytes, gel polymer electrolytes (GPEs), which are also referred to as quasi‐solid‐state electrolytes, provide a good compromise between high ionic conductivity, low flammability, and mechanical stability.^[^ ^3^ ^]^ GPEs typically consist of three components: a polymer matrix, an organic solvent or plasticizer, and a lithium salt. As a drawback, GPEs are usually characterized by a low lithium mobility and transference number (*t*_Li_^+^ < 0.5) and consequently are more susceptible to polarization phenomena that eventually limit the power delivery during battery discharge.^[^ ^3^, ^4^ ^]^

Single‐ion conducting polymer electrolytes (SIPEs) conductors were introduced in the early 90s, with the aim to increase the lithium mobility *t*_Li_^+^. SIPEs are polyelectrolytes, where the anions are covalently tethered to the polymer backbone and the counter lithium ions are the only free‐mobile species.^[^ ^5^, ^6^, ^7^, ^8^ ^]^ The combination of SIPEs and GPEs offers materials which simultaneously exhibit high lithium transference number, high ionic conductivities at room temperature, and good mechanical robustness. This allows lithium metal battery operation even at room temperature.^[^ ^9^ ^]^ Among the different polymer chemistries, lithium sulfonylimide‐based monomers dominate the actual SIPE development because their expanded conjugated structure can delocalize the negative charge in a relatively efficient manner and hence decrease the binding energy with Li‐ions.^[^ ^10^, ^11^, ^12^ ^]^ In particular, lithium sulfonamide methacrylate monomer (LiMTFSI), developed by Shaplov and Armand, has shown superior properties compared with the styrene monomer version (LiSTFSI).^[^ ^11^, ^13^, ^14^ ^]^ Thus, the conductivity of the methacrylic TFSI‐based electrolytes is higher, by at least a factor of two than the styrenic version.^[^ ^15^ ^]^ The versatile LiMTFSI methacrylic monomer has been included in different SIPE polymer compositions including networks, random and block copolymers. and homopolymer blends.^[^ ^6^, ^7^, ^9^, ^16^, ^17^, ^18^, ^19^, ^20^, ^21^, ^22^ ^]^

In the last years, the demand for printable electrolytes has grown due to the emergence of new battery technologies including microbatteries and shape conformable batteries. In contrast, the fast development of additive manufacturing and 3D printing technologies are offering a range of advantages including freedom design for complex geometries, rapid prototyping, cost‐effectiveness, reduced material waste, and more importantly, the potential for customization.^[^ ^23^ ^]^ Some examples of additive manufacturing methods have already been demonstrated to print materials for batteries.^[^ ^24^ ^]^ For instance, Schubert et al. designed a film by UV‐printing of an ionic liquid gelled in a methacrylate‐based polymer matrix for all organic batteries.^[^ ^25^ ^]^ Other printed GPEs, UV‐induced, were presented by Furukawa and Chung and co‐workers, in which they printed a GPE by photopolymerization of methacrylamides, polyvinyldene fluoride, and LiCl dissolved in polyethylene glycol.^[^ ^26^ ^]^ In the second iteration they developed a printed GPE based on acrylates and polysiloxanes in conventional carbonates and ether electrolytes, always via UV‐induced printing.^[^ ^27^ ^]^ Besides UV‐induced printing, direct ink writing (DIW) is an extrusion‐based technique that is lately increasingly used for extrusion of gels and quasi‐solid polymer electrolytes for energy‐based devices.^[^ ^28^ ^]^ DIW has some advantages such as the wide range of materials employed, the simplicity of the process and low cost.^[^ ^29^ ^]^ The development of high performance GPEs which can be processed by DIW are actively being searched.^[^ ^30^, ^31^, ^32^ ^]^

The goal of this work was to develop DIW printable single‐ion gel polymer electrolytes for lithium batteries. For this purpose, we investigated the synthesis of polymeric nanoparticles (NPs) functionalized with a lithium sulfonamide methacrylate monomer by emulsion polymerization. Recently, we showed that polymeric NPs (size between 100 and 200 nm) can be formulated as solid electrolytes with good ionic conductivity.^[^ ^33^ ^]^ Herein, we progress the synthesis towards smaller size NPs (< 100 nm) through a one step emulsion polymerization, where different parameters such as the surfactant concentration and the lithium sulfonamide co‐monomer ratio were optimized. SIPEs with small NPs and different plasticizers commonly used in gel polymer electrolytes, such as carbonates and sulfolane, were prepared. These soft solid electrolyte materials were subsequently characterized and their 3D printability was investigated by DIW printing, including detailed rheological analysis. Once the printing parameters were identified, solid electrolyte layers were directly printed as separators onto battery electrodes such as lithium metal. The electrochemical characteristics of the solid electrolytes and the resultant printed batteries were finally evaluated.

## 2 Results and Discussion

### 2.1 Synthesis and Characterization of Lithium Sulfonamide Functionalized Methacrylic Crosslinked Polymer NPs

**Figure 1:**
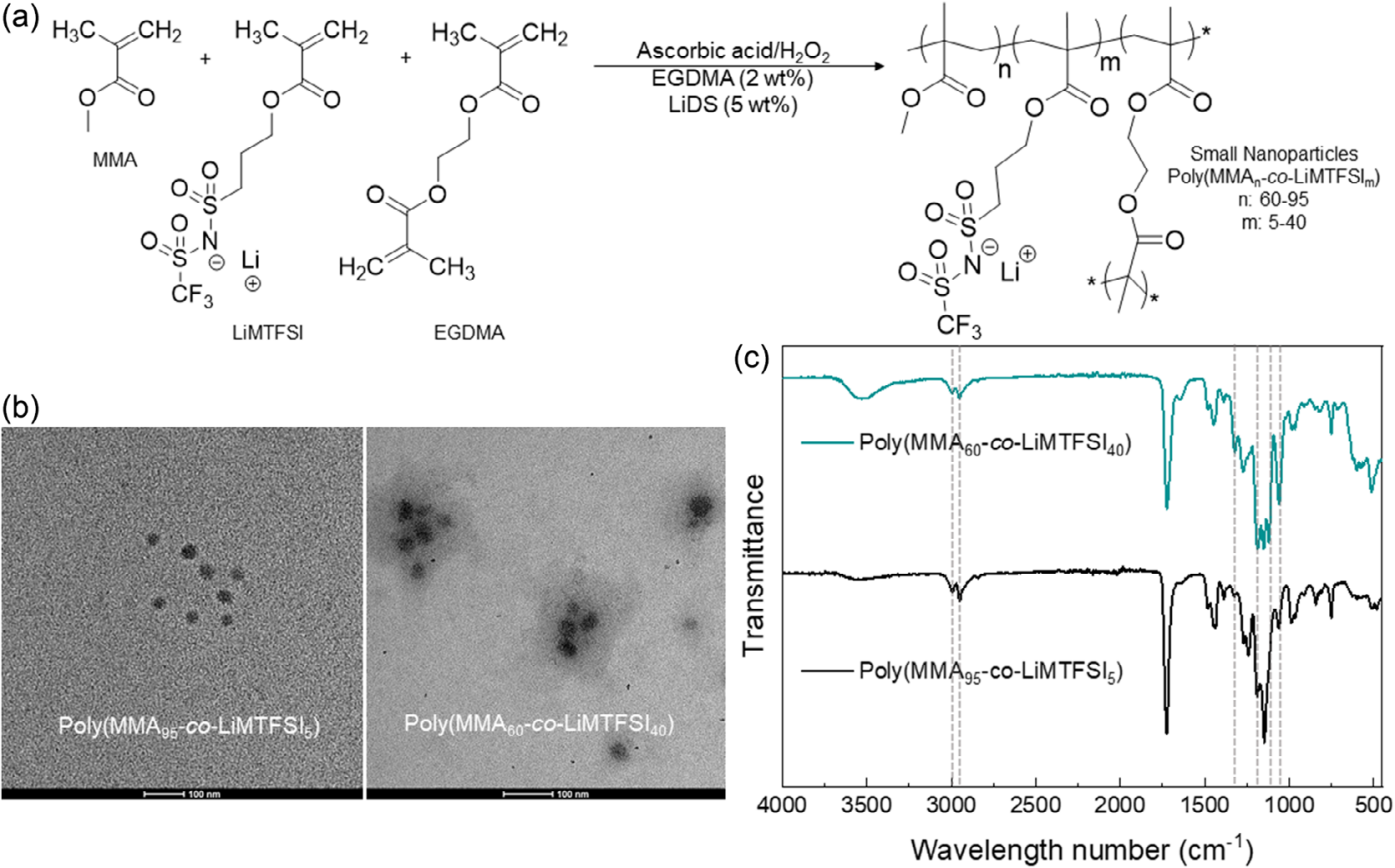
a) Representative scheme of lithium sulfonamide and methyl methacrylate NPs synthesized by emulsion polymerization. b) TEM images (notice that bar length is 100 nm, which length varies for different LiNPs) and c) FTIR spectra comparison between two single‐ion NP compositions: Poly(MMA_95_ *‐co*‐LiMTFSI_5_) and Poly(MMA_60_ *‐co*‐LiMTFSI_40_).

Lithium‐functionalized polymer NPs were synthesized by emulsion copolymerization of lithium sulfonamide methacrylate monomer (LiMTFSI) and methyl methacrylate (MMA), in the presence of a crosslinker (**Figure** **1**a). In our previous work, we used a surfactant free method, which allowed us to synthesize NPs of a large size of around 200 nm. In order to synthesize very small NPs, we introduced 5 wt% of lithium dodecyl sulfate as surfactant. The lithium version of the commonly used surfactant was chosen in order to avoid the presence of extra cations, such as sodium, or undesirable cation exchange processes. The random polymerization procedure was carried out in water at 70 °C with a 10 wt% solids content for 6 h, employing ascorbic acid and hydrogen peroxide as redox initiator system and EGDMA as a crosslinker. The ratio between LiMTFSI and MMA co‐monomer was varied at three different polymer compositions (Table S1, Supporting Information), Poly(MMA_n_ *‐co*‐LiMTFSI_m_), being *n* and *m* the molar% fractions of each monomer. As a result of the emulsion polymerization process, transparent polymer latexes were obtained indicating the small size of the polymer NPs. After purification by dialysis, the latexes were freeze‐dried and polymer NPs were obtained as fine powders in high weight conversion (>85 wt%).

Several characterizations were carried out to elucidate the chemical composition, size and shape of the polymer NPs. First, quantitative ^13^C‐NMR was carried out for the quantification of lithium functionalization and the co‐monomer ratio in the polymeric NPs, as shown in Figure S1–S3, Supporting Information. The integration of the NMR signals of C═O at 175.4 ppm with respect to CF_3_ at 120.5 ppm were compared to determine the molar ratio between the monomeric units of MMA and LiMTFSI. The polymer NPs showed higher experimental compositions than the monomer feed ratio with following compositions based on mol%: Poly(MMA_95_ *‐co*‐LiMTFSI_5_), Poly(MMA_83_ *‐co*‐LiMTFSI_17_) and Poly(MMA_60_ *‐co*‐LiMTFSI_40_) (see Table S1, Supporting Information). This NMR measurement may be more sensitive to the functionalization on the surface of the NPs than in the interior of the crosslinked matrixes. The higher amount of lithium monomeric units in the surface may be due to a matter of ionic stability of the polymeric NPs.

Inductively coupled plasma mass spectroscopy (ICP‐MS) was also carried out for all polymeric NPs to confirm the incorporation and the quantity of the lithium sulfonamide functionalized monomer. Poly(MMA_95_ *‐co*‐LiMTFSI_5_) showed 2.1 mg Li^+^/g Np, which corresponds to a 1.7 mol% of LiMTFSI in the material, Poly(MMA_83_ *‐co*‐LiMTFSI_17_) presented a 5.7 mol% and Poly(MMA_60_ *‐co*‐LiMTFSI_40_) a 18.3 mol%. This technique successfully confirmed the presence and quantity of Li^+^ in the materials. LiMTFSI mol% for all lithium sulfonamide functional nanoparticles (LiNPs) is lower than the theoretical proposed one, as shown in Table S1, Supporting Information. This difference is possibly due to a diffusion of the sulfonamide ionic monomer into the aqueous phase, since it is more likely for the monomer to prefer a further stable aqueous environment. To sum up, ICP‐MS and ^13^C‐NMR techniques successfully confirmed the presence of Li^+^ in the NPs, while the ICP‐MS contributed with the information of the overall lithium sulfonamide composition and the NMR being a bit more sensitive to the surface one.

FTIR spectroscopy also revealed the successful copolymerization between LiMTFSI and MMA confirming the chemical structure of the polymeric NPs. Figure 1c presents the comparison spectra between the Poly(MMA_60_ *‐co*‐LiMTFSI_40_) and Poly(MMA_95_ *‐co*‐LiMTFSI_5_) NPs containing 5 and 40 mol% of Li^+^ ratio. The absorption bands observed in both spectra with similar intensity at 1725 cm^−1^ belongs to the stretching vibration of C═O from the methacrylate group. The absorption bands at 1322, 1121, and 1060 cm^−1^ correspond to the S=O stretching from the sulfonamide moieties, peaks that are notably increased when Poly(MMA_60_ *‐co*‐LiMTFSI_40_) spectrum is analyzed as compared to Poly(MMA_95_ *‐co*‐LiMTFSI_5_). The peak at 1187 cm^−1^ belongs to the stretching vibration of C–F from the CF_3_ groups in the NPs, again being stronger in Poly(MMA_60_ *‐co*‐LiMTFSI_40_) spectrum due to the higher lithium monomer contribution.

All in all, FTIR confirms the chemical composition of the polymeric NPs which was previously quantified by ^13^C‐NMR.

As mentioned in the introduction, our goal here is to synthesize polymer NPs of very small sizes (<100 nm) compared to our previous work (200 nm).^[^ ^33^ ^]^ First, dynamic light scattering (DLS) was employed to estimate the hydrodynamic volume of the polymeric NPs. The three synthesized copolymer latexes showed very small sizes of around 25 nm with a low polydispersity without a clear influence of the composition. To further measure the size and the shape, the NPs were analyzed by TEM. As observed in Figure 1b, spherical NPs with very small sizes between 22 and 30 nm were obtained. After analysis with ImageJ software, Poly(MMA_95_ *‐co*‐LiMTFSI_5_) NPs showed an average diameter of 21.5 ± 3.1 nm, Poly(MMA_83_ *‐co*‐LiMTFSI_17_) a size of 28.4 ± 5.9 nm and Poly(MMA_60_ *‐co*‐LiMTFSI_40_) a size of 32.1 ± 6.4 nm showing a size trend dependence of the copolymer composition. Figure S4, Supporting Information, shows extra TEM images of all LiNPs, including for the Poly(MMA_83_ *‐co*‐LiMTFSI_17_) LiNPs that are not included in Figure 1.

Finally, thermal characterization of the polymer NPs was carried out through dynamic scanning calorimetry (DSC) and thermogravimetric analysis (TGA). Figure S5a, Supporting Information, presents the TGA results, showing a thermal stability up to 250 °C with a 5 wt% degradation, with no considerable difference between the different compositions. DSC results can be observed in Figure S5b, Supporting Information, where a single glass transition (*T*_g_) is observed at ≈127 °C for Poly(MMA_95_ *‐co*‐LiMTFSI_5_), ≈112 °C for Poly(MMA_83_ *‐co*‐LiMTFSI_17_) and ≈104 °C for Poly(MMA_60_ *‐co*‐LiMTFSI_40_). The higher the composition in MMA in the NPs, the higher the *T*_g_ as expected for this type of cross‐linked random copolymers.

### 2.2 Preparation of Lithium Single‐Ion Conducting Gel Polymer Electrolytes

**Figure 2:**
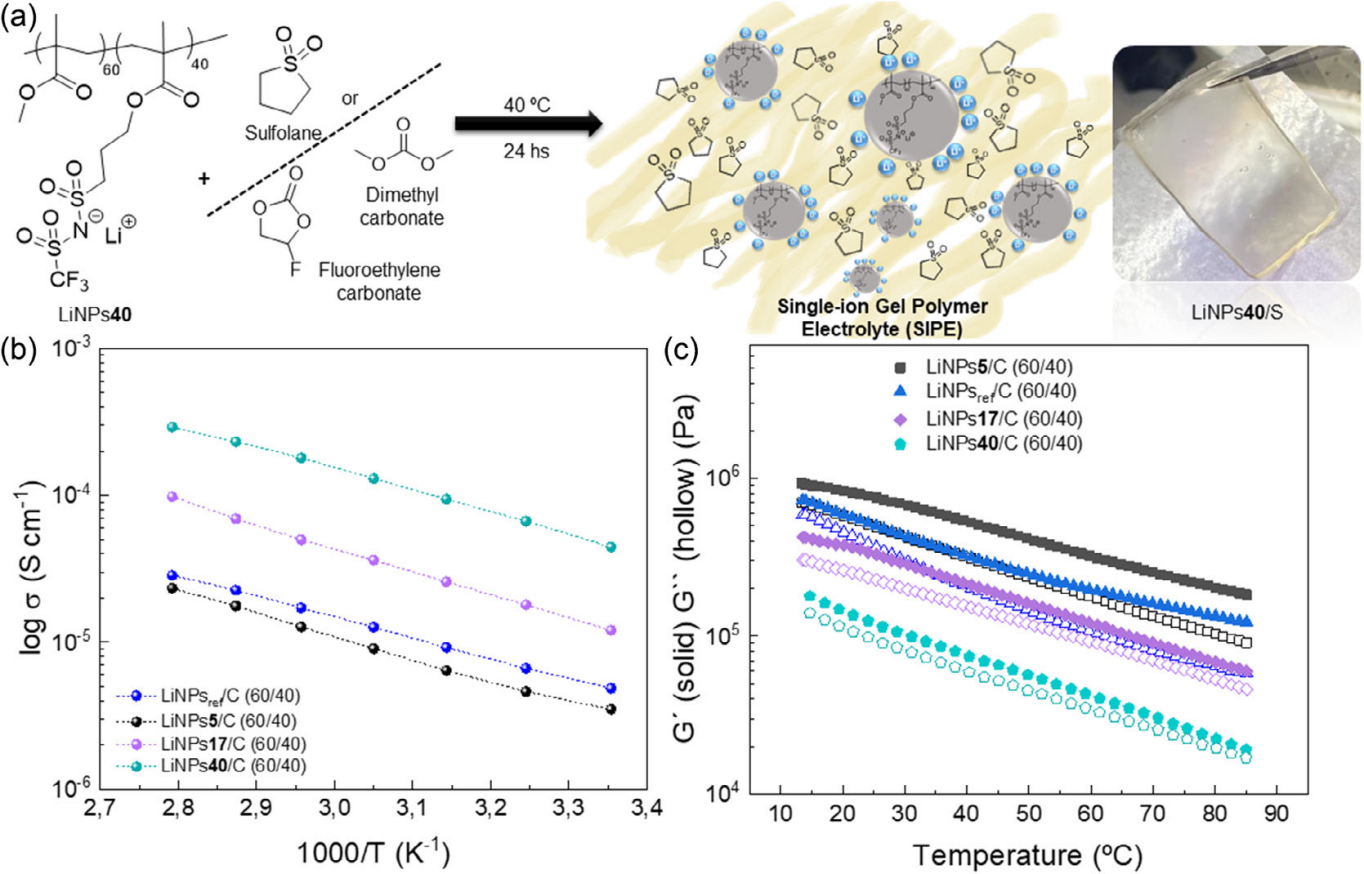
a) Single‐ion conducting lithium polymer electrolyte preparation employing LiNPs and sulfolane/carbonates, b) Ionic conductivity versus temperature and c) Dynamic mechanical analysis (DMA) of carbonates.

Solid polymer electrolytes were simply prepared by mixing the lithium sulfonamide functionalized polymer NPs with a plasticizer in a given ratio. **Figure** **2**a shows the representative scheme of the polymer electrolyte preparation employing sulfolane as plasticizer. As a result a flexible polymer film was obtained (see Figure 2a). The ratio between the polymer NPs and the plasticizer was varied from 50 to 60 wt%, which was the optimized composition identified in our previous work. Polymer electrolytes were also prepared using commonly used carbonates in battery electrolytes as plasticizers. In this case, we employed a mixture of dimethyl carbonate (DMC) and fluoroethylene carbonate (FC) (3:1) which is known as a good electrolyte composition.^[^ ^35^ ^]^ Table S2, Supporting Information, shows the detailed compositions and definitions. It is worth to note that the only source of lithium in these polymer electrolytes comes from the lithium sulfonamide attached to the polymer NP without the addition of extra lithium salt. This is expected to produce a SIPE.

The transport and mechanical properties of the carbonates‐based SIPEs employing 60 wt% single‐ion NP and 40 wt% carbonates were characterized as shown in Figure 2. Figure 2b shows the ionic conductivity versus temperature of polymer electrolytes prepared with the four different NP compositions, including a comparison of NP size effect. The polymer electrolytes prepared with the small size NPs presents a conductivity of 9.9 × 10^−5^ S cm^−1^ at 85 °C, a value more than three times higher than the same SIPE prepared with the big 100 nm single‐ion polymer NPs (≈2.9 × 10^−5^ S cm^−1^ at the same temperature). This huge difference of ionic conductivity between electrolytes prepared under the same lithium composition using NPs with different sizes 25 vs 100 nm justifies our synthetic efforts towards small size NPs. We anticipate that a small size of the NPs means large NP surface area, and therefore, a greater exposure of the sulfonamide lithium within the more mobile plasticized regions. The effect of the NP composition and the presence of different amount of lithium sulfonamide functionalities, i.e., LiNPs5/C, LiNPs17/C and LiNPs40/C, is also compared in Figure 2b. At 85 °C, the ionic conductivity of the SIPEs shows a higher value when the amount of LiMTFSI increase in the polymeric NPs. Thus, LiNPs5/C shows an ionic conductivity of 2.3 × 10^−5^ S cm^−1^, LiNPs17/C a 9.9 × 10^−5^ S cm^−1^ one and LiNPs40/C the highest at 2.9 × 10^−4^ S cm^−1^at the maximum temperature measured. This data shows more than an order of magnitude increase in ionic conductivity when 40 mol% of the lithium monomer is present in the copolymer versus 5 mol%. However, the increased ion conductivity cannot be merely due to an increase in the number of Li^+^ ions as this is only a factor of 8 while the conductivity increase is more than ten times. Thus, we established that ionic conductivity is superior in the case of polymer electrolytes based on small size NPs with high Li^+^ sulfonamide content.

The mechanical properties of the gel polymer electrolytes based on NPs with varying size and composition were investigated and presented in Figure 2c. The storage modulus measured by rheology of the SIPEs employing LiNPs5/C was the highest (4.1 × 10^6^ Pa) followed by the LiNPs17/C SIPE (1.6 × 10^6^ Pa) and finally LiNPs40/C based‐SIPE (5.8 × 10^5^ Pa) at 50 °C. The tendency found was similar to what was observed for ionic conductivity along the temperature range; the higher the lithium content in the NPs surface, the lower the storage modulus. It can be hypothesized that higher lithium concentration generates a solvation shell given by the interaction with carbonates, which increases the softness of the gel.

### 2.3 Extrusion 3D Printing of SIPEs

Taking into account the ionic conductivities and mechanical properties, polymer electrolytes based on the Poly(MMA_60_ *‐co*‐LiMTFSI_40_), LiNPs 50 wt% and 50 wt% of plasticizer were selected for 3D printing studies. Polymer electrolytes were prepared using two different plasticizers, cyclic carbonates, and sulfolane. In both cases, transparent films were obtained as observed in Figure S6, Supporting Information, for LiNPs40/C and LiNPs40/S. While carbonate‐based SIPE presented a jelly and viscous appearance (Figure S6a, Supporting Information), sulfolane‐based SIPE showed great manageability and a free‐standing membrane appearance (Figure S6b, Supporting Information). Then, ionic conductivity of both was measured employing electrochemical impedance spectroscopy (EIS) between 25 and 95 °C (Figure S7, Supporting Information). As expected, the conductivity of cyclic carbonate gel polymer electrolytes at 25 °C of LiNPs40/C (3.3 × 10^−5^ S cm^−1^) is superior to the sulfolane ones LiNPs40/S (1.7 × 10^−5^ S cm^−1^), though this difference becomes small when temperature increases.

**Figure 3:**
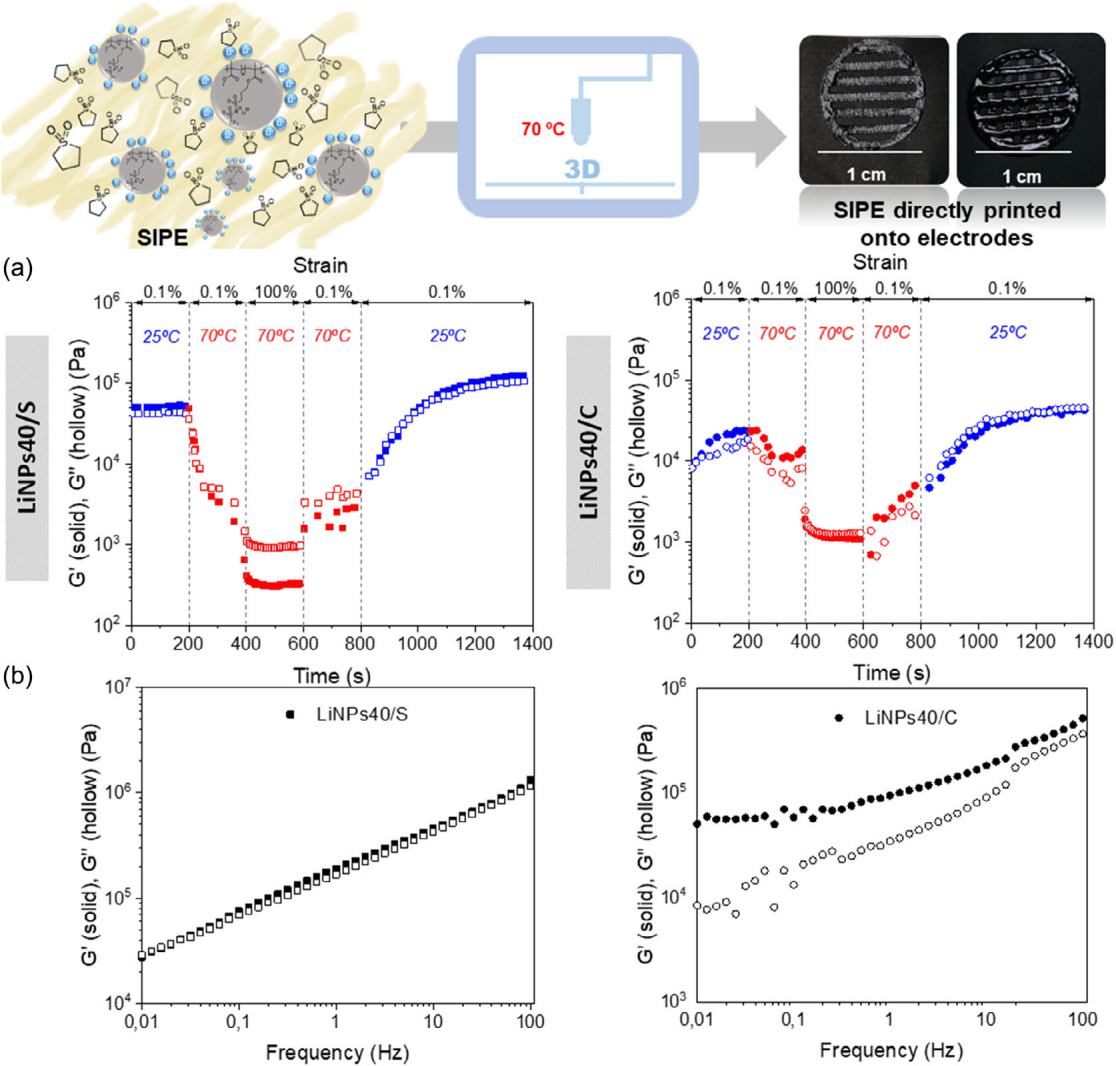
Representative scheme of SIPEs DIW onto some classical electrodes for lithium battery. Rheology experiments of sulfolane and carbonates based‐SIPEs. a) Dynamic step‐strain amplitude tests at printing and recovery temperature conditions and b) frequency sweeps of LiNPs40/S and LiNPs40/C.

The dynamic rheological properties of inks are important parameters for melting‐based extrusion printing.^[^ ^36^ ^]^ For DIW, inks should behave as yield‐stress fluids with a marked shear thinning during flow and a fast recovery of their elastic properties after shear. For this reason, a rheological study was carried out in order to evaluate the use of our polymer electrolytes as inks, as well as their printability.^[^ ^37^ ^]^ Dynamic step‐strain amplitude tests at low (25 °C) and high (70 °C) temperatures were performed to determine time‐dependent behaviors with respect to moduli (**Figure** **3**a). In the first step at low strains (0.1%) and room temperature (25 °C), which mimics the steady state of the ink inside the cartridge before printing, both sulfolane based‐SIPE, LiNPs40/S and carbonates‐based‐SIPE, LiNPs40/C exhibit a solid‐like behavior (G′ > G″). Then, in a second step, the effect of increasing the temperature of the ink without varying the applied strain was measured at 70 °C (printing temperature). It was observed a decreased of G′ and G″ in both cases, which was even more pronounced in the case of LiNPs40/S. In a third step, after applying a high strain (100%) at 70 °C, LiNPs40/S exhibited a liquid‐like behavior (G″ > G′) proving the flowing of the ink. In contrast, LiNPs40/C did not exhibit a clear dominance of G″ over G′, connoting that at that determined strain and temperature the ink did not show a good liquid‐like behavior for extrusion printing. In the fourth step, the properties of the ink after removing the high applied strain (0.1%) and keeping the high temperature (70 °C) were determined, showing a slight increase of G′ and G″. Finally, the mechanical properties of the ink after extrusion, that is at low strains (0.1%) and room temperature (25 °C), were registered. Results pointed out a recovery of the initial values of G′ and G″ and the solid‐like behavior of LiNPs40/S after the extrusion conditions, whereas LiNPs40/C need much longer times.

Figure 3b presents frequency sweeps experiments, which both sulfolane and carbonate‐ based‐SIPEs present a gel‐like behavior at the steady state after extrusion. Concerning strain‐dependence properties of SIPEs after extrusion, LiNPs40/S showed a linear viscoelastic region (Figure S8, Supporting Information), where G′ and G″ are independent of strain, up to 1% yield strain (crossover point of G′ and G″). In the case of LiNPs40/C, the linear viscoelastic region is enlarged up to >100% yield strain. As observed, LiNPs40/S presents a lower critical strain, around 1% strain, while LiNPs40/C needs a higher oscillation strain (≈100% strain) to start flowing, suggesting that sulfolane‐based SIPE generates greater fluency on the printing ink.

All in all, rheology experiments indicate that sulfolane NP polymer electrolytes show a superior extrusion printing behavior than carbonates based‐SIPE. For this reason, LiNPs40/S was 3D printed layer by layer via direct ink writing directly onto classic battery electrodes, forming an electrolyte separator layer. Figure 3 shows some images of micro‐structured SIPEs directly printed onto the battery electrodes of one layer (left picture) and four layers printed onto the electrode (right picture), being each of them of around 100 μm. After printing experiments, some thermal characterization of LiNPs40/S was carried out in order to evaluate their stability. As observed in Figure S9, Supporting Information, the sulfolane based‐SIPE is thermally stable up to 200 °C with a melting point at 3 °C.

### 2.4 Electrochemical Characterization of Single‐Ion NP/Sulfolane Polymer Electrolytes for Lithium Battery Applications

**Figure 4:**
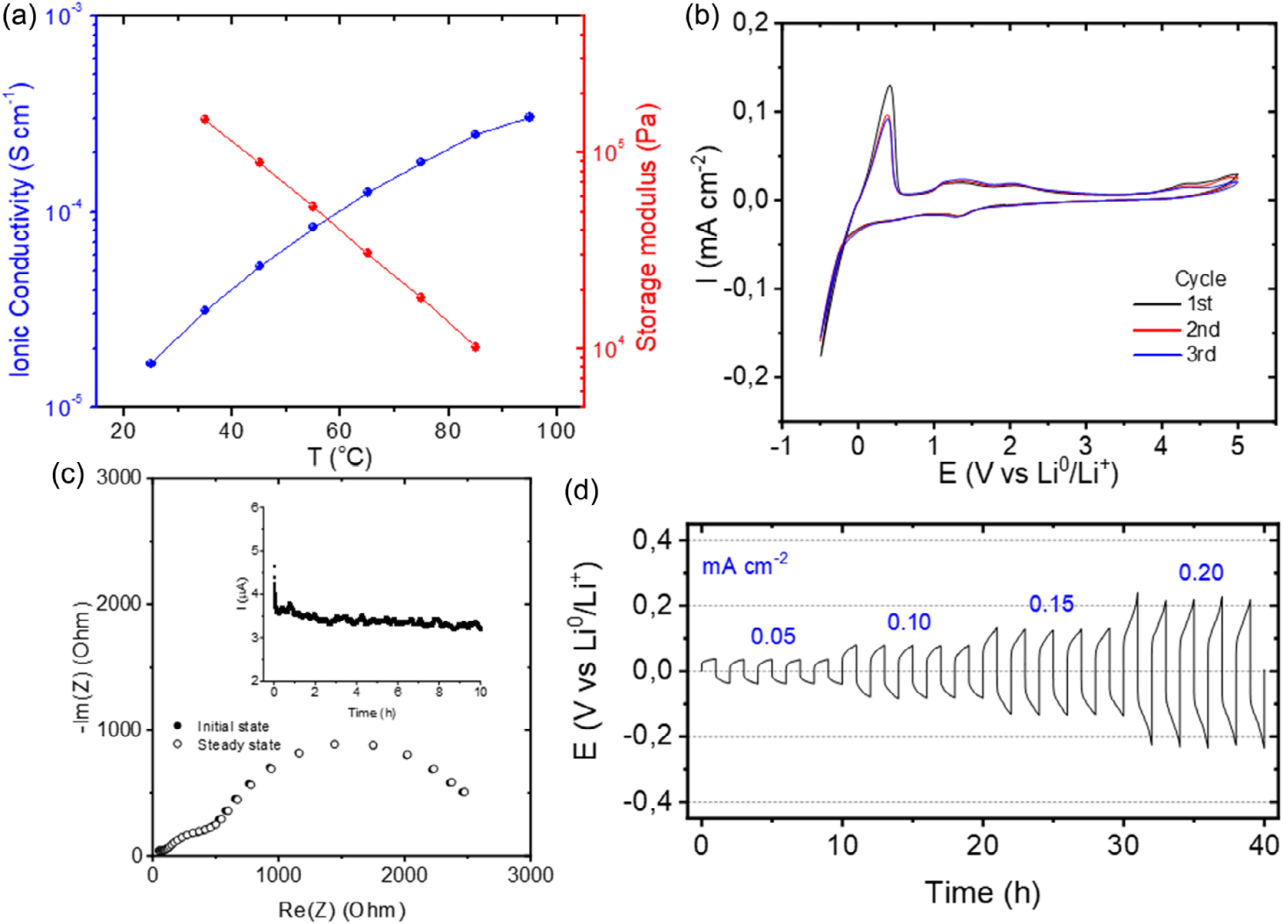
a) Electrochemical characterization of sulfolane based‐SIPE by ionic conductivity, b) plating/stripping, c) AC and DC‐measurements for lithium ion transfer number and d) potential as a function of time for symmetrical lithium cells.

The electrochemical properties of the printed LiNPs40/S polymer electrolytes were investigated in order to assess its potential for lithium battery applications. **Figure** **4**a shows the ionic conductivity and the modulus of the sulfolane based‐SIPE. The values of ionic conductivity in the order of 10^−4^ S cm^−1^ are appropriate for solid state battery operation. The ionic conductivity increases with temperature showing a classical Arrhenius behavior. Next, the electrochemical stability window of the sulfolane‐based SIPE was studied by cyclic voltammetry at 50 °C (Figure 4b). The anodic breakdown potential of the sample was found to be higher than 5 V versus Li^+^/Li^0^, enabling its potential use in high‐voltage batteries involving NMC, LMO, or LCO. Generally, the anodic stability of gel electrolytes is determined by the electrochemical stability of the solvent. Here, sulfolane is known to present a high dielectric constant (43.4) and high oxidation potential (>6.35 V) which accounts for this excellent electrochemical stability of our gel electrolyte.^[^ ^34^, ^38^, ^39^ ^]^ Regarding the cathodic scans, a highly reversible redox processes were observed between −0.5 and 0.48 V versus Li^+^/Li^0^, which is evidently associated with lithium plating/stripping and confirms the efficient transfer of lithium ions through the polymer electrolyte.

The lithium transfer number was determined to confirm that the gel material behaves as a single lithium ion conductor (Figure 4b). Using the Bruce–Vincent–Evans method, the lithium‐ion transference number (*t*_Li_^+^) was calculated for the sulfolane NP polymer electrolyte at 50 °C. A *t*_Li_^+^ value of 0.67 was obtained which confirms a high fraction of ionic conductivity due to the movement of the lithium cations. It is worth to note that although this *t*_Li_^+^ value is not as high as other lithium single‐ion conductors (*t*_Li_^+^  > 0.8) it is higher than most polymer electrolytes (*t*_Li_^+^  < 0.3). The lower than unity *t*_Li_^+^ may be due to the intrinsic inaccuracy of the measurement method or may suggest bulk movement of NPs contributing an anionic current.

Lithium transport properties of the polymer electrolyte were further evaluated in a lithium symmetric cell at 50 °C (Figure 4d). The current was varied between 0.05 to 0.2 mA cm^−2^ with a polarization period of 1 h and repeated for five cycles. Such experiments allow to assess two major criteria of a solid electrolyte: 1) lithium transport properties, which needs to be sufficient to sustain the current density applied, and 2) ability to form a highly conductive solid electrolyte interphase (SEI) under the defined cycling conditions (also influences its electrochemical stability towards lithium metal). A failure of an electrolyte to meet one of these two criteria will lead to poor cycling performance. The single‐ion SIPE shows a low overpotential (<200 mV at 0.2 mA cm^−2^) and a distinct plateau, indicating that this system exhibits very good lithium transport properties without any sign of short‐circuit, enabling its application at these current densities.

**Figure 5:**
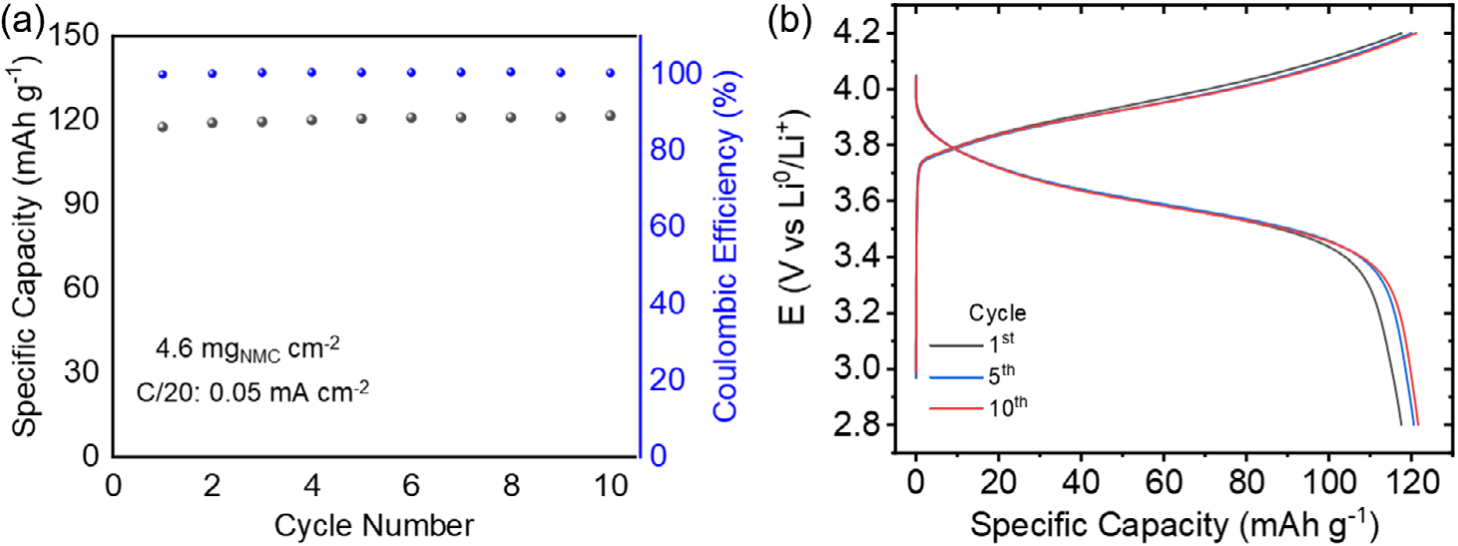
a) Specific discharge capacity vs cycle number and b) voltage profiles of Li|LiNPs40/S|NMC(111) full cell at 50 °C using a current density of 0.05 mA cm^−2^ (C/20).

Finally, the printable solid electrolyte was also evaluated in Li|LiNPs40/S|NMC(111) battery cells at 50 °C (see **Figure** **5**a). A high voltage cathode material was selected due to the excellent electrochemical stability of the solid electrolyte. As observed in the graph, a current density of 0.05 mA cm^−2^ was used to cycle the cell at C/20 reaching a capacity of 121.6 mAh g^−1^ (0.56 mAh cm^−2^) and a Coulombic efficiency close to 100%. As the cycling goes on, the cell shows a very stable performance and even slightly improving the capacity during the first ten cycles as observed in Figure 5b. By analyzing the voltage profiles, a small and stable polarization is observed, which indicates that the solid electrolyte has not been damaged during the cycling. Overall, the preliminary battery results demonstrate the potential of printed LiNPs40/S for solid‐state high‐voltage lithium batteries.

## 3 Conclusions

In this work, we developed DIW printable single‐ion gel polymer electrolytes for use in solid‐state lithium batteries. The polymer electrolytes were based in synthesized methacrylic polymeric NPs functionalized with a lithium sulfonamide group. The synthesis of the lithium sulfonamide methacrylic NPs was successfully carried out by emulsion polymerization leading to very small NPs of sizes less than 30 nm as shown by TEM analysis.

We investigated the preparation of SIPEs combining NP of varying lithium sulfonamide content and two different plasticizer systems, carbonates and sulfolane. The ionic conductivity of the three SIPEs showed values between 10^−4^ and 10^−5^ S cm^−1^. The polymer electrolyte showed the highest ionic conductivities and robust mechanical properties when smaller size NPs with higher lithium sulfonamide co‐monomer ratio are employed. Then rheology experiments indicated that sulfolane plasticized polymer electrolytes showed an optimum and superior extrusion printing behavior than carbonate ones.

Finally, a sulfolane‐based polymer electrolyte was 3D printed layer‐by‐layer via direct ink writing directly onto classic battery electrodes, forming an electrolyte separator layer. The electrochemical characterizations of the polymer electrolytes show that they present excellent properties (ionic conductivity, lithium transfer number, electrochemical stability window, and cyclability in lithium symmetrical cells) with the potential to be used in lithium batteries. All in all, this article presents a promising new family of printable single‐ion polymer NP gel electrolytes which will be investigated for lithium full batteries fabrication in the near future.

## 4 Experimental Section

4.1

4.1.1

##### Materials

Methyl methacrylate (MMA, 99%), ethylene glycol dimethacrylate (EGDMA, 98%), ascorbic acid (99%), lithium dodecyl sulfate (LiDS, 98.5%), sulfolane (99%), propylene carbonate (98%), dimethyl carbonate (99%), and fluoroethylene carbonate (99%) were obtained from Sigma Aldrich. Hydrogen peroxide 30 v v^−1^% was bought from Fisher. All reagents were employed with no further purification.

##### Synthesis of Lithium Sulfonamide Functionalized Methacrylic NPs

Lithium‐functionalized polymer NPs were synthesized through emulsion polymerization of LiMTFSI and MMA from a modified method reported in a previous work.^[^ ^9^ ^]^ This one‐pot synthesis was carried out in water at 70 °C in 10 wt% solids content, during 6 h, employing ascorbic acid and hydrogen peroxide as redox initiator system, EGDMA as crosslinker and lithium dodecyl sulfate as surfactant.

In a 100 mL flask, 35 g of MilliQ water and 0.25 g of lithium dodecyl sulfate (0.9 mmol) were purged with N_2_ during 20 min at 50 °C. Separately, in three different vials, were pre‐dissolved and purged with N_2_: a) between 0.5 and 2.5 g of LiMTFSI (1.5, 2.9, and 7.2 mmol) and 0.114 g of ascorbic acid (0.65 mmol) in 5 g of MilliQ water; b) between 2.5 and 4.5 g of MMA (25, 40 or 45 mmol) and 0.1 g of EGDMA (0.52 mmol); and c) 0.065  mL of H_2_O_2_ in 5 g of MilliQ water. After the purging, the three separated solutions were slowly incorporated at a flow rate of 1 mL min^−1^ to the 100 mL flask with an increment of the temperature until 70 °C.

After polymerization, the polymeric NP dispersions were filtered with an 80 μm nylon mesh to remove solids (coagulated NPs). The amount of coagulum was calculated based on the total solid content of the dispersion and was less than 1 wt%. The NPs were then purified with MilliQ water during 5 days at 25 °C employing dialysis tubes with a molecular weight cutoff of 14 000 Da and finally freeze‐dried with a Telstar LyoQest‐55 Lyophilizer at −55 °C and 0.055 mbar during 3 days. The obtained fine white powder of each class of NP was dried under reduced pressure at 70 °C for 24 h and transferred into an argon‐filled glovebox.

##### SIPEs Preparation

SIPEs were obtained by mechanical stirring of the lithium sulfonamide functionalized NPs in the desired plasticizer in a given ratio. Two plasticizer systems were used, one based on a combination of carbonates, dimethyl carbonate: fluoroethylene carbonate (3:1 wt ratio), and the other was sulfolane, the high boiling point solvent currently being considered for lithium batteries.^[^ ^34^ ^]^

##### Characterization Techniques

^13^C Nuclear Magnetic Resonance (^13^C‐NMR) spectra were recorded in a Bruker Avance DPX 300 at 75.5 MHz of resonance frequency, using dimethylsulfoxide (DMSO) as solvent at room temperature. NPs were properly dispersed in the deuterated solvent before the measurements.

The hydrodynamic diameter of the LiNPs were measured by dynamic light scattering (DLS) using a Malvern Zetasizer Nano ZS. A drop of reaction mixture (≈0.1 mL of the dispersion in water) was diluted with 1 mL of MilliQ water employing disposable polystyrene cuvettes DTS0012. The intensity average was measured at 25 and 173 °C backscatter angle by using dynamic light scattering Malvern ZetaSizer Nano‐S instrument equipped with a 633 nm red laser.

The diameter and the morphology of the dried LiNPs were measured by transmission electron microscopy (TEM) employing a TECNAI G2 20 TWIN (200 kV). The samples were deposited on copper grids and left to dry at room temperature.

ICP‐MS was carried out in order to determine the presence and quantity of Li^+^ in the single‐ion NPs. An ICPMS Agilent series 8900 was employed, Ar gas was used as the plasma formation source. Conditions of the analysis: radio frequency power 1550 W, plasmogen gas flow 15 L min^−1^, auxiliar gas flow 0.9 L min^−1^, nebulizer gas flow 1 L min^−1^.

Fourier transform infrared (FTIR) spectra of the NPs were measured on a Bruker Alpha II spectrophotometer employing Platinum ATR module with diamond window.

The thermal stability of the lithium NPs and their corresponding SIPEs were investigated by thermogravimetric analysis (TGA) performed on a TGA Q500 from TA Instruments. The samples were heated at 10 °C min^−1^ under a N_2_ atmosphere from room temperature to 800 °C. Differential scanning calorimetry was employed to detect the *T*_g_ of the different single‐ion polymer NPs by measurements on a DSC Q2000 from TA Instruments. The DSC scans were performed at heating and cooling rates of 10 °C min^−1^ from −60 to 200 °C.

Temperature sweep experiments were performed in a stress‐controlled Anton Paar Physica MCR101 rheometer, employing an 8 mm disposable parallel plate geometry. Experiments were carried out with constant strain of 1% at a constant frequency of 1 Hz, varying temperature from 15 to 85 °C for carbonates and from 15 to 100 °C for sulfolane (linear range of viscoelasticity of the materials). The processability properties were evaluated by dynamic step‐strain amplitude tests by varying the strain between 0.1% and 100% at 25 and 70 °C, using an AR‐G2 rheometer (TA instruments) equipped with a stainless‐steel parallel plate geometry of 20 mm diameter and a gap of 1 mm. The elastic modulus (G′) and loss modulus (G″) of the NPs after the dynamic step‐strain amplitude tests were determined by strain and frequency sweeps. Strain sweeps from 0.01 to 100 Hz were performed at 1 Hz and 25 °C. Frequency sweeps from 0.01 and 100 Hz were performed at 1% strain and 25 °C.

Electrochemical impedance spectroscopy (EIS) was employed to measure the ionic conductivity of the lithium functionalized NPs and the SIPEs in an Autolab 302 N potentiostat galvanostat at different temperatures, between 25 and 95 °C. An equilibration time of 20 min was required before every measurement. The samples were placed between two stainless steel electrodes (surface area of 0.5 cm^2^). Measurements were recorded in the 100 kHz to 1 Hz range, with 10 mV amplitude. Cell assembly was carried out inside a glovebox to avoid any humidity that may increase the ionic conductivity of the materials. Lithium NPs and the SIPEs were maintained inside a glovebox to prevent humidity.

##### 3D Direct Ink Writing (DIW) of SIPEs

3D DIW of SIPEs based on carbonates and sulfolane was carried out with a 3D‐Bioplotter (Developer Series, EnvisionTEC, Gladbeck, Germany). The printed patterns were designed with the software Autodesk Inventor 2019. The printing process of sulfolane‐based NPs was performed at 70 °C, maximum pressure of 5 bar, 0.5 mm s^−1^ speed, and using needles with an inner diameter of 0.6 mm. The layer‐by‐layer printing was performed with a waiting time between layers of 30 s. The printing of carbonate‐based NPs was performed at 60 °C, maximum pressure of 3 bar, 1 mm s^−1^ speed, and using needles with an inner diameter of 0.4 mm. The layer‐by‐layer printing was performed with a waiting time between layers of 10 s.

##### Battery Tests

Fresh Li||Li symmetrical cells were also employed to characterize lithium transference number and ramp tests. For the lithium‐ion transference number (*t*_Li_^+^) measurement, after 10 h of resting, EIS was used in the range of 1 MHz–0.1 Hz with a perturbation of 40 mV and a polarization potential of 80 mV for 4 h. The Bruce–Vincent–Evans method was applied to determine $t_{Li^{+}}$ according to the following equation

(1) $$t_{{\text{Li}}^{+}}=\frac{I_{s}\left(\Delta V-I_{0}R_{0}\right)}{I_{0}\left(\Delta V-I_{s}R_{s}\right)}$$

where Δ*V* is the applied potential across the cell, *I* is the current, *R* is the interfacial resistance, and the subscripts s and 0 are the steady‐state and initial values, respectively. Plating‐stripping was also evaluated at different current densities in the range of 0.05–0.20 mA cm^−2^ for 1 h step both charge and discharge during five cycles.

High‐voltage cathode was prepared by mixing NMC (111), C_65_ and LiNPs40/S in a weight ratio of 60/10/30 employing acetone as solvent with the aid of a Speed Mixer at 2000 rpm for 15 min. The resultant slurry was coated with an automatic doctor blade (Neurtek) and leave it drying at room temperature. Afterwards, the cathode was further dried at 60 °C under high vacuum to remove the traces of acetone before any characterization leading to 4.6 mg_NMC_ cm^−2^ mass loading.

Li|LiNPs40/S|NMC(111) full cells were assembled by sandwiching LiNPs40/S (160 μm thick) in between Lithium metal anode (13 mm in diameter) and NMC cathode (8 mm in diameter). Finally, the cell was cycled at 50 °C at C/20 (0.05 mA cm^−2^).

## Conflict of Interest

The authors declare no conflict of interest.

## Supporting information

Supplementary Material

## Data Availability

The data that support the findings of this study are available on request from the corresponding author. The data are not publicly available due to privacy or ethical restrictions.
