# Supplementary material for: Printable Single‐Ion Polymer Nanoparticle Electrolytes for Lithium Batteries
Source: Small Sci. 2024 Jan 14;4(3):2300235. doi: 10.1002/smsc.202300235 (PMC11935232; doi:10.1002/smsc.202300235)
Supplement: Supplementary file 1 — Supplementary Material [file SMSC-4-2300235-s001.pdf]

Printable Single-ion Polymer Nanoparticle Electrolytes for Lithium Batteries

Antonela Gallastegui[*], *Rafael del Olmo, Miryam Criado-Gonzalez, Jose Ramon Leiza, Maria Forsyth, David Mecerreyes*[*]

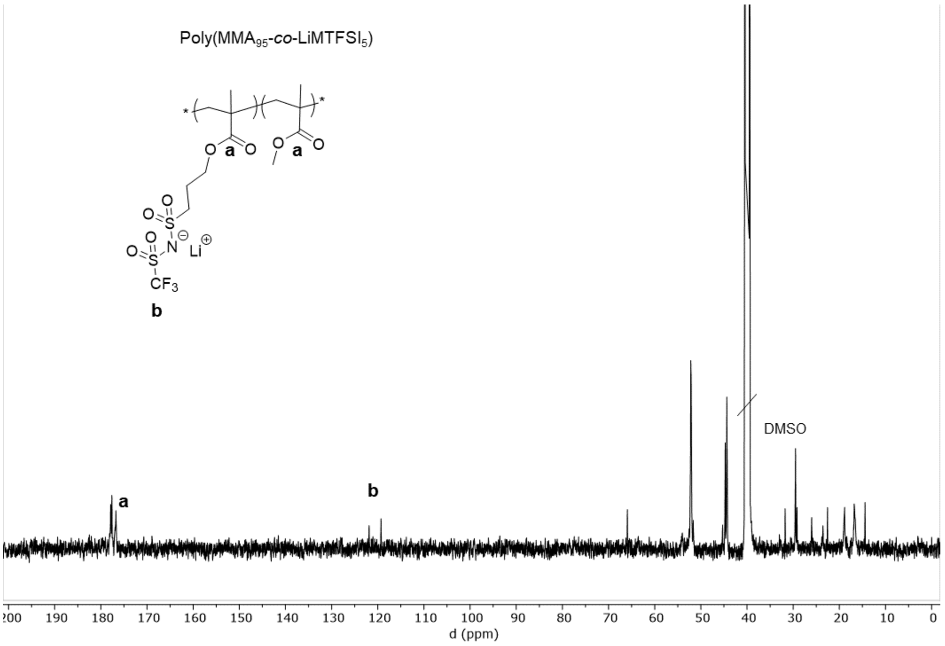

Figure S1. ^{13}C-NMR spectrum of Poly(MMA$_{95}$-*co*-LiMTFSI$_5$) in *d*-DMSO.

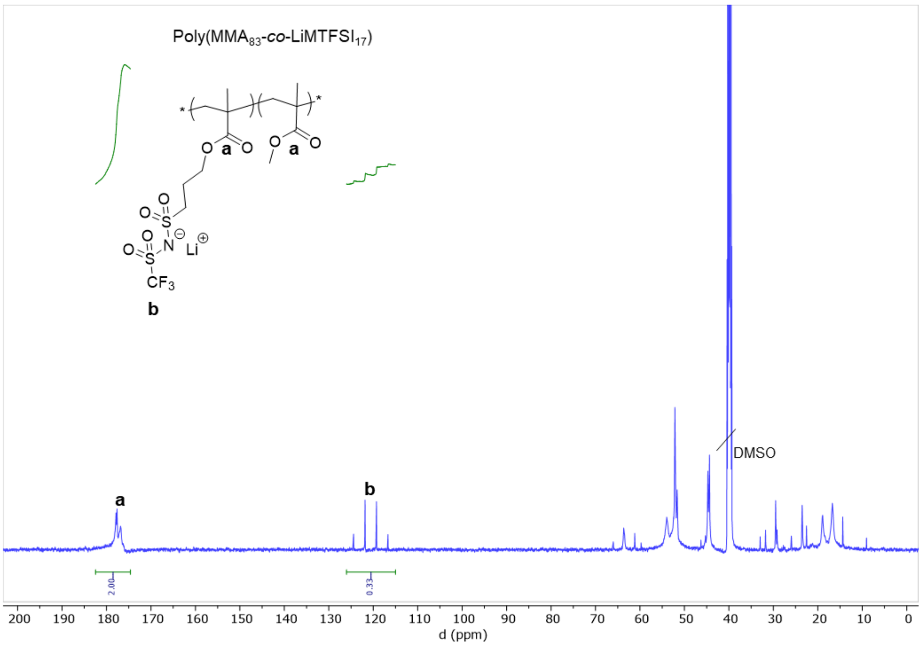

Figure S2. ^{13}C-NMR spectrum of Poly(MMA$_{83}$-*co*-LiMTFSI$_{17}$) in *d*-DMSO.

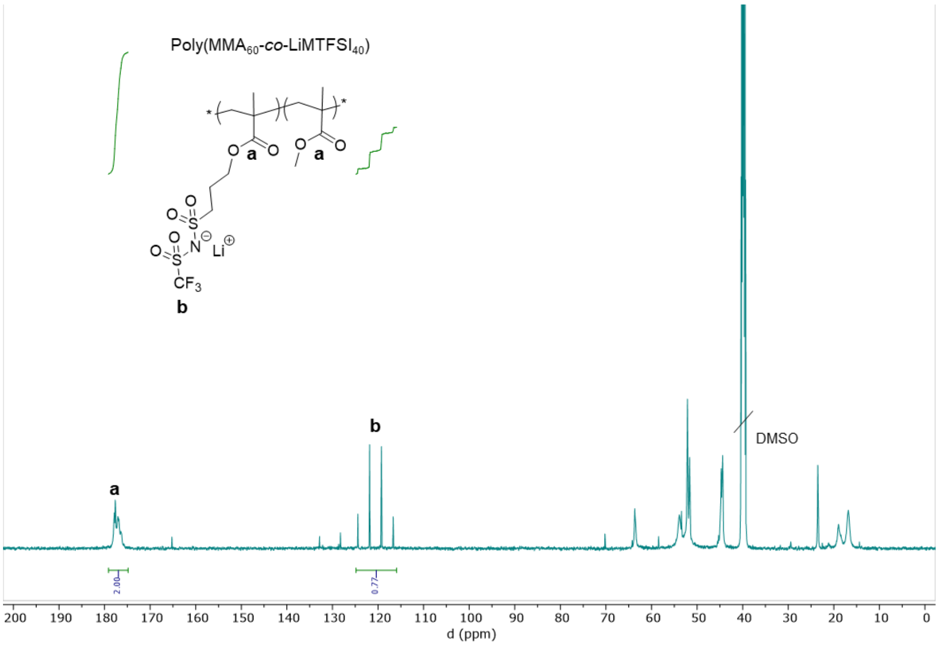

Figure S3. ^{13}C-NMR spectrum of Poly(MMA$_{60}$-*co*-LiMTFSI$_{40}$) in *d*-DMSO.

Table S1. Single-ion NP characterizations.

NPs	Conv.[a]	Size (intensity)[b]	PDI [b]	Mol% th[c]	ICP-MS[d]	Mol% exp[e]	Mol% exp[f]
**Poly(MMA$_{95}$-*co*-LiMTFSI$_{5}$)**	85%	24.1 nm	0.18	3.5	2.1	2.8	5.1
**Poly(MMA$_{83}$-*co*-LiMTFSI$_{17}$)**	82%	27.8 nm	0.16	6.8	3.4	5.7	16.9
**Poly(MMA$_{60}$-*co*-LiMTFSI$_{40}$)**	85%	27.0 nm	0.29	22.4	8.7	18.3	40.3

[a] Conversion by weight; [b] Size and polydispersity (PDI) determinations by DLS; [c] theoretical mol% of the NPs [d] ICP-MS results given by Li^+ mg per Np g; [e] ICP-MS results given by mol%, [f] Mol% given by ^{13}C NMR in deuterated DMSO.

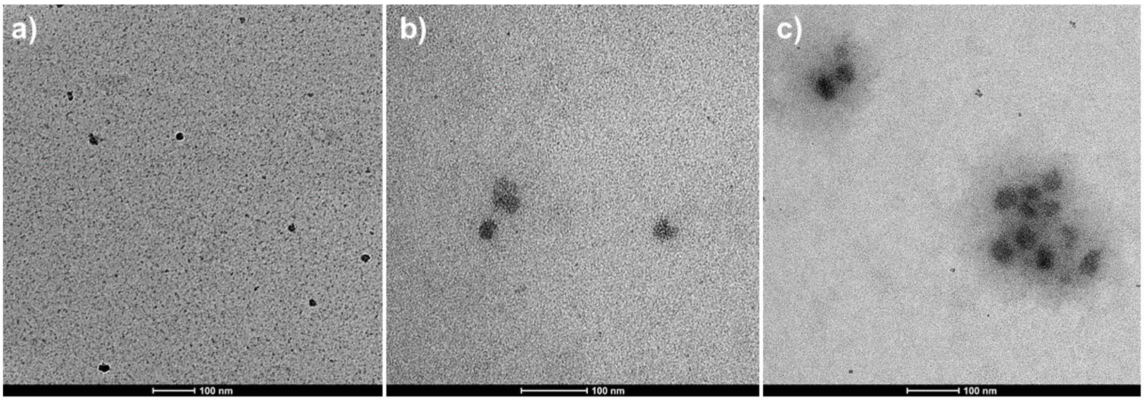

Figure S4. Extra TEM images of the LiNPs: Poly(MMA$_{95}$-*co*-LiMTFSI$_{5}$) (a), Poly(MMA$_{83}$-*co*-LiMTFSI$_{17}$) (b) and Poly(MMA$_{60}$-*co*-LiMTFSI$_{40}$) (c).

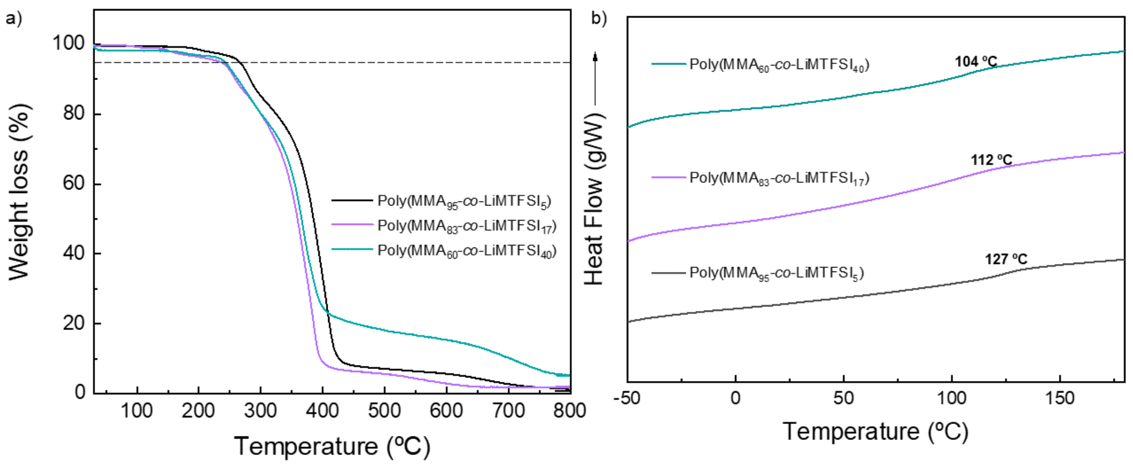

Figure S5. Thermal gravimetric analysis (TGA) (a) and Dynamic scanning calorimetry (DSC) (b) of all LiNPs.

Table S2. Single-ion polymer nanoparticle gel electrolytes (SIPEs) designation.

LiNP	Carbonate	Sulfolane
**LiNPs reference 100 nm**	$LiNPs_{ref}$/C	$LiNPs_{ref}$/S
**Poly(MMA$_{95}$-*co*-LiMTFSI$_{5}$)**	LiNPs**5**/C	LiNPs**5**/S
**Poly(MMA$_{83}$-*co*-LiMTFSI$_{17}$)**	LiNPs**17**/C	LiNPs**17**/S
**Poly(MMA$_{60}$-*co*-LiMTFSI$_{40}$)**	LiNPs**40**/C	LiNPs**40**/S

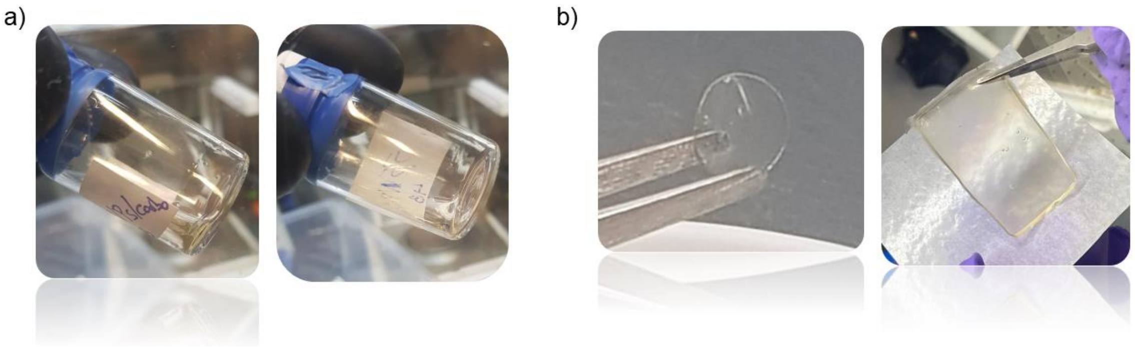

Figure S6. Pictures of Carbonates (a) and sulfolane based-SIPE (b) based on LiNPs**40**.

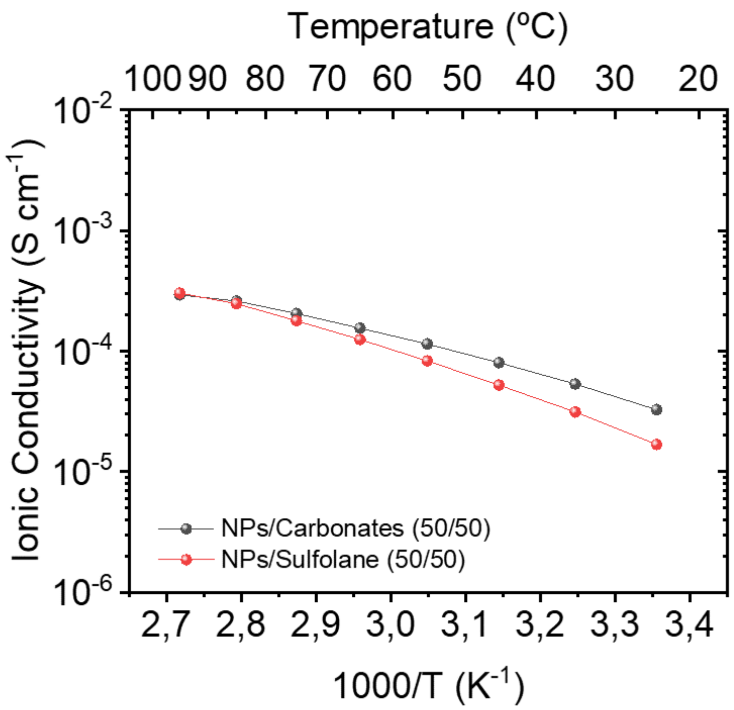

Figure S7. Ionic conductivity determined by EIS for carbonates and sulfolane-based SIPE.

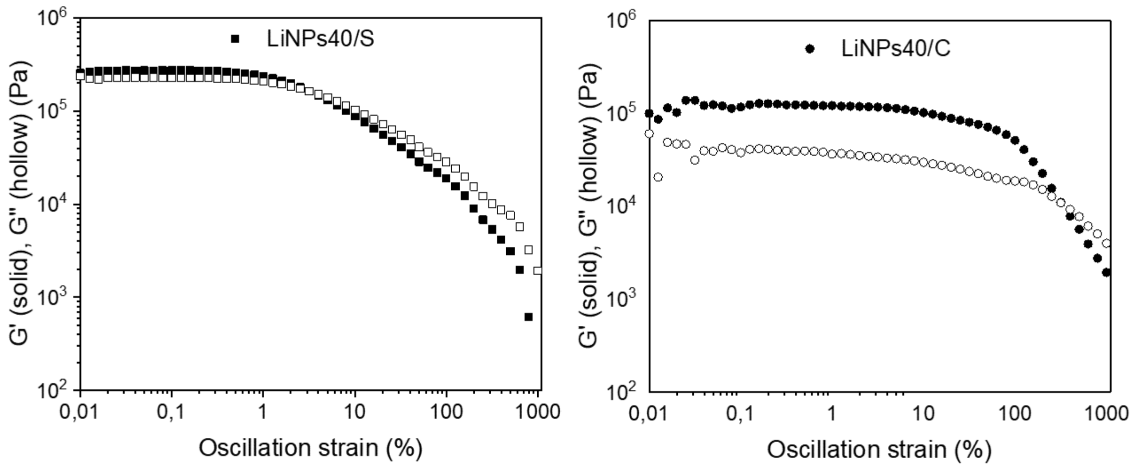

Figure S8. Oscillatory stress sweeps of LiNPs**40**/S and LiNPs**40**/C.

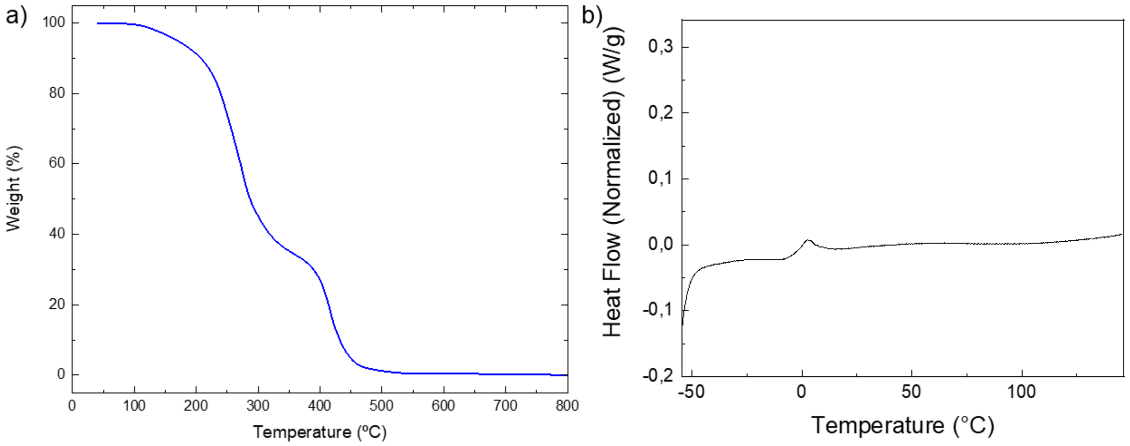

Figure S9. Thermal characterization of sulfolane based-SIPE by TGA (a) and DSC (b).
